# An electronic family health history tool to identify and manage patients at increased risk for colorectal cancer: protocol for a randomized controlled trial

**DOI:** 10.1186/s13063-019-3659-y

**Published:** 2019-10-07

**Authors:** Karen M. Goldstein, Deborah A. Fisher, R. Ryanne Wu, Lori A. Orlando, Cynthia J. Coffman, Janet M. Grubber, Tejinder Rakhra-Burris, Virginia Wang, Maren T. Scheuner, Nina Sperber, Santanu K. Datta, Richard E. Nelson, Elizabeth Strawbridge, Dawn Provenzale, Elizabeth R. Hauser, Corrine I. Voils

**Affiliations:** 1 Durham Center of Innovation to Accelerate Discovery and Practice Transformation, Durham Veterans Affairs Health Care System, 508 Fulton Street, Durham, NC 27705 USA; 2 Division of General Internal Medicine, Duke University School of Medicine, Durham, NC USA; 3 Division of Gastroenterology, Department of Medicine, Duke University School of Medicine, Durham, NC USA; 4 Durham Cooperative Studies Program Epidemiology Center, Durham Veterans Affairs Health Care System, Durham, NC USA; 5 Center for Applied Genomics and Precision Medicine, Duke University School of Medicine, Durham, NC USA; 6 Department of Biostatistics and Bioinformatics, Duke University School of Medicine, Durham, NC USA; 7 Department of Population Health Sciences, Duke University School of Medicine, Durham, NC USA; 8 Division of Medical Genetics, University of California at San Francisco, San Francisco, CA USA; 9 Division of Hematology-Oncology, San Francisco Veterans Affairs Health Care System, San Francisco, CA USA; 10 Health Services Research, Management and Policy, University of Florida College of Public Health and Health Professions, Gainesville, FL USA; 11 IDEAS Center, VA Salt Lake City Healthcare System, Salt Lake City, UT USA; 12 Division of Epidemiology, University of Utah School of Medicine, Salt Lake City, UT USA; 13 William S Middleton Memorial Veterans Hospital, Madison, WI USA; 14 Department of Surgery, University of Wisconsin, Madison, WI USA

**Keywords:** Risk assessment, Family history, Cancer screening, Colorectal cancer

## Abstract

**Background:**

Colorectal cancer is the fourth most commonly diagnosed cancer in the United States. Approximately 3–10% of the population has an increased risk for colorectal cancer due to family history and warrants more frequent or intensive screening. Yet, < 50% of that high-risk population receives guideline-concordant care. Systematic collection of family health history and decision support may improve guideline-concordant screening for patients at increased risk of colorectal cancer. We seek to test the effectiveness of a web-based, systematic family health history collection tool and decision support platform (MeTree) to improve risk assessment and appropriate management of colorectal cancer risk among patients in the Department of Veterans Affairs primary care practices.

**Methods:**

In this ongoing randomized controlled trial, primary care providers at the Durham Veterans Affairs Health Care System and the Madison VA Medical Center are randomized to immediate intervention or wait-list control. Veterans are eligible if assigned to enrolled providers, have an upcoming primary care appointment, and have no conditions that would place them at increased risk for colorectal cancer (such as personal history, adenomatous polyps, or inflammatory bowel disease). Those with a recent lower endoscopy (e.g. colonoscopy, sigmoidoscopy) are excluded. Immediate intervention patients put their family health history information into a web-based platform, MeTree, which provides both patient- and provider-facing decision support reports. Wait-list control patients access MeTree 12 months post-consent. The primary outcome is the risk-concordant colorectal cancer screening referral rate obtained via chart review. Secondary outcomes include patient completion of risk management recommendations (e.g. colonoscopy) and referral for genetic consultation. We will also conduct an economic analysis and an assessment of providers’ experience with MeTree clinical decision support recommendations to inform future implementation efforts if the intervention is found to be effective.

**Discussion:**

This trial will assess the feasibility and effectiveness of patient-collected family health history linked to decision support to promote risk-appropriate screening in a large healthcare system such as the Department of Veterans Affairs.

**Trial registration:**

ClinicalTrials.gov, NCT02247336. Registered on 25 September 2014.

**Electronic supplementary material:**

The online version of this article (10.1186/s13063-019-3659-y) contains supplementary material, which is available to authorized users.

## Background

Colorectal cancer (CRC) is the fourth most commonly diagnosed cancer in the United States, with 140,788 new cases and 52,396 deaths in 2015 [1]. Screening by several modalities has been shown to reduce death from CRC in average-risk individuals [2–7] and is recommended by several national organizations [8]. CRC screening via colonoscopy or stool testing is recommended for average-risk individuals beginning at the age of 45 years by the American Cancer Society [9] and beginning at 50 years by the United States Preventive Services Task Force [10] and a Multi-society Task Force [8]. However, different screening modalities and/or frequencies are recommended for patients above population-level risk (hereafter referred to as increased risk), such as those with a personal or family history of colorectal neoplasia or adenomatous polyps [8].

Risk assessment using family health history (FHH) permits tailoring of CRC screening to an individual’s risk level. While collection of FHH is incorporated into multiple clinical guidelines [11, 12], it is rarely used in clinical practice as part of a standardized risk assessment [13]. Challenges to FHH use in clinical practice include physician time constraints during office visits, lack of provider awareness of FHH collection standards, failure of the medical record to support adequate collection and documentation of FHH collection, and provider lack of confidence in identifying patients at increased risk for CRC [14–16]. Subsequently, although 3–10% of the United States population [17] is thought to be at increased risk for CRC and, thus, meets criteria for more intensive screening and management, < 50% receive this recommended care [18–20].

Systematic FHH collection and provision of clinical decision support (CDS) can improve accuracy of identification and appropriate prevention management of patients at increased risk for disease. For example, Qureshi et al. found that, compared to usual care, systematic FHH collection identified previously undetected primary care patients who were at increased risk for cardiovascular disease (4.8% vs 0.3%) [21]. Systematic FHH collection can also improve appropriate use of strategies for increased-risk management. Orlando et al. found that the use of a web-based FHH risk assessment tool increased the proportion of increased-risk management strategies being delivered appropriately to increased risk patients from 10.5% to 71.8% [22]. Similarly, Scheuner et al. found that a cancer family history toolkit administered in primary care practices increased family history cancer documentation and increased guideline-concordant referral for genetic consultation more than twofold [23].

Integrated health systems, such as the Veterans Administration (VA), can benefit from systematic approaches to FHH collection as they often internally refer patients for procedures and counseling indicated by patient risk. Currently, the VA does not have a system-wide approach to the routine collection and documentation of FHH in the medical record. This lack of systematic documentation can result in poor FHH documentation. For example, at one VA healthcare system, the documentation of FHH for any cancer was 26.6% of patients seen [23]. Another study of 500 consecutive patients at a VA hospital revealed that 7% of fecal occult blood test orders by primary care providers (PCPs) were inappropriate and should have been referred for colonoscopy instead due to a personal or FHH that increased risk of CRC [24]. A standardized FHH collection tool supported by decision support for patients and providers could provide needed structure to improve risk-appropriate CRC management [23].

To address the pressing clinical need for improved risk-identification and risk-appropriate CRC screening, we are conducting a cluster randomized controlled trial (RCT) to evaluate the feasibility and effectiveness of implementing a web-based, systematic FHH collection and decision support platform (MeTree) [25] in VA primary care practices. We will evaluate the effect of the intervention on PCPs’ workflow and health economic outcomes to inform eventual implementation more broadly. To that end, our primary aims are: (1) to determine whether FHH collection via MeTree improves identification of patients at increased risk for CRC; and (2) to evaluate whether patient and provider decision support improves risk-appropriate PCP referrals for, and patient uptake of, CRC screening/surveillance and referral for genetic counseling. Our secondary aims are: (1) to assess experience with clinical decision support reports and effects on primary care provider workflow via qualitative interviews; and (2) to conduct budget-impact analyses of implementing FHH collection and decision support in the VA.

## Methods

### Study setting and design

We are conducting this RCT at two sites: the Durham VA Health Care System in Durham, NC, including three associated primary care clinics, and the William S. Middleton Memorial Veterans Hospital in Madison, WI. All enrolled patients are veterans receiving care at one of these medical facilities. Institutional review boards at the Durham VA Health Care System and the University of Wisconsin-Madison (the institutional review board of record for the Madison VA hospital) approved this study.

This study is a two-arm, cluster-randomized, type I hybrid implementation-effectiveness trial that aims to test the effectiveness of a clinical intervention (i.e. family history risk assessment with decision support documents) while obtaining data to inform potential future implementation [26]. Enrolled PCPs are randomized to have their patients complete MeTree either upon enrollment (immediate arm) or 12 months later (wait-list control arm). PCP randomization is stratified by site (Durham vs Madison) and provider type (physician versus advanced practice provider [physician assistant or nurse practitioner]) in case there are differences in extent of knowledge about FHH in guideline-concordant care or use of the information in clinical practice. We aim to sub-stratify by age within provider sub-groups (40–49 years vs 50–64 years) to address the potential for intervention effects that differ across age groups given that population-based screening begins at the age of 50 years. We chose a 12-month wait-list control arm as the comparator group to ensure that all patients receive the benefit of risk-appropriate screening. The primary outcome for this study is risk-appropriate CRC screening referral at 12 months. Secondary outcomes include patient completion of CRC screening referral and risk-appropriate genetic consultation, economic outcomes, and barriers and facilitators to intervention use (see Table 1 for additional details of study activities by time point). This trial is registered at clinicaltrials.gov (NCT02247336) (see Additional file 1 for additional trial registration items). Checklist for standard protocol items are listed in Additional file 2.

**Table 1:** Study activities by time point (SPIRIT Table)

		Study time point			
	Provider recruitment T_− 2_	Patient screening T_− 1_	Baseline T_0_	0–30 days post-baseline T_1_	12 months post-baseline T_2_
Provider recruitment					
Contact eligible providers	X				
Info sessions at clinic	X				
Informed Consent	X				
Randomization	X				
Patient recruitment					
Mailing recruitment letters		X			
Telephone screen		X			
Informed consent			X		
Randomization revealed			X		
Intervention (MeTree)					
Immediate arm				X	
Delayed arm					X
Assessments					
Baseline survey			X		
Exit survey – immediate				X	
Exit survey – delayed					X
12-month survey (all)					X
Chart abstraction (all)					X

### Study population and recruitment

#### Primary care provider recruitment and randomization

**Fig. 1:**
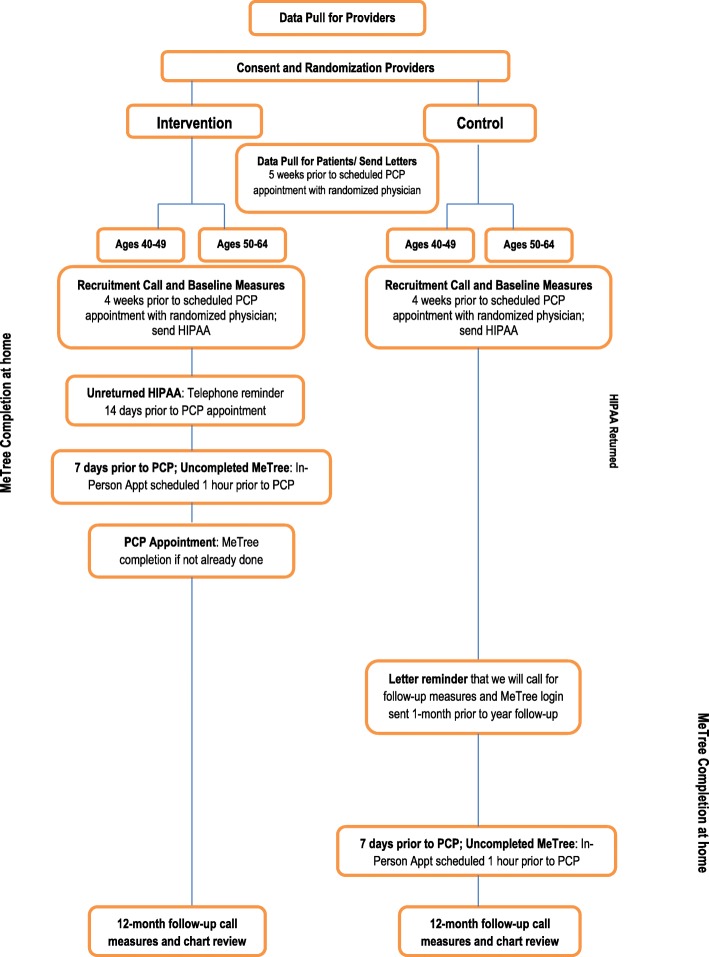
Study flow

Provider inclusion/exclusion criteria are listed in Table 2. To recruit providers, we attend staff meetings to introduce the study; we do not provide education regarding FHH or CRC during these meetings. PCPs can consent to participate at the meeting or later via email. We use a rolling recruitment strategy to capture any newly hired providers who wish to participate. Providers enroll via email to the site PI in Durham (KG) and a study team member in Madison. The study coordinator (ES) enters consented providers into a study database for randomization assignment. Randomization is at the provider level to avoid contamination by a provider having patients in both study arms (see Fig. 1). Using SAS version 9.4 software, the study statistician developed a randomization scheme for allocating providers to the immediate or wait-list control arm and within stratification groups of provider type (advanced practice provider [nurse practitioner or physician assistant] vs physician). The resulting randomization assignment list was imported into the study’s tracking database. As providers consent to participate, they are assigned to the arm that was randomly associated with the next vacant slot on the randomization list for their provider type. Within the stratification groups, providers are allocated in block sizes (block size is unknown to everyone but study statisticians).

**Table 2:** Eligibility criteria and method of ascertainment

	Criteria	Method
Primary care provider		
Inclusion	Physician, nurse practitioner, or physician assistant	Administrative & Phone screen
	Working in one of the targeted primary care clinics	Administrative & Phone screen
	At least one half-day of primary care clinic per week	Administrative & Phone screen
	Patient panel size large enough to enroll 12–13 patients	Administrative & Phone screen
Patient participant		
Inclusion	Assigned to one of the enrolled PCPs	Administrative & Phone screen
	At least one primary care appointment in the 18 months before enrollment	Administrative & Phone screen
	Upcoming PCP appointment with assigned PCP	Administrative & Phone screen
	Aged 40–64 years	Administrative & Phone screen
	No previous history of CRC (V10.05, V10.06, ICD-9153, 154), inflammatory bowel disease (555.0, 555.1, 555.2, 555.9, 556.0), or adenomatous polyps (indicated in pathology report)	Administrative & Phone screen
Exclusion	Endoscopy within the previous 3 years	Administrative & Phone screen
	Plans to relocate or leave the VA healthcare system in the next 12 months	Phone Screen
	More than 2 errors on a validated 6-item screener for cognitive impairment	Phone screen
	Non-English speaking	Phone screen
	No knowledge of FHH of first- and second-degree predecessor relatives, even if adopted	Phone screen
	Concurrent enrollment in a competing research study (related to colon cancer)	Phone screen

#### Patient recruitment

Patient eligibility is determined using a combination of electronic medical record (EMR) data and telephone screening (see Table 2). We identify patients of enrolled PCPs with an upcoming primary care appointment (at least six weeks out) using EMR appointment schedules. We mail patients a recruitment letter and informational brochure about MeTree. Patients may opt out of the study by calling a toll-free number; otherwise, study personnel may contact potential patient participants by telephone to explain the study and assess interest. During the telephone contact, study staff verify eligibility and willing patients provide verbal consent (see Additional file 3 for informed consent language).

During the recruitment call, consented patients complete a baseline telephone survey (see Table 3) about health screening history and a computer proficiency assessment [28]. After completing the survey, we inform patients of their randomization assignment, determined by whether their PCP was randomized to the immediate versus wait-list control arm. Once randomized, patients remain in their original treatment assignment arm even if they change providers. To be enrolled in the study and thus included in analyses, patients must complete MeTree to determine their CRC risk level. We expect a lower MeTree completion rate for patients in the wait-list control arm compared to the immediate arm because of the planned 12-month delay between baseline survey and MeTree assessment in the control arm. Therefore, we plan to enroll approximately 100 more patients in the control arm than in the immediate arm to enable us to meet our targeted sample size of *n* = 250 completed MeTree assessments in each arm (see the “Statistical power and sample size” section).

**Table 3:** Survey measures by study time point

Measures	Baseline survey	12-month survey	Exit survey
Demographics	X		
Quality of life (Euro-QoL [27])	X	X	
Personal history of genetic testing	X	X	
Personal history of health screening^a^	X	X	
Computer proficiency (CPQ-12 [28])	X		
Experience with MeTree			X
Topics discussed with PCP			X

^a^CRC, breast cancer, ovarian cancer, lung cancer, diabetes mellitus, and cardiovascular disease

#### Blinding

Enrolled providers are not informed of their study arm at enrollment. However, we do alert them that their patients will start receiving the intervention either shortly after enrollment or 12 months later. Patient receive their treatment arm assignment immediately after completing the baseline survey. Blinding of outcome assessors cannot be completely ensured as described below under “Measures,” but attempts are made to preserve blinding as much as possible.

### Study procedures

#### Immediate arm

A few weeks before beginning patient enrollment, we provide PCPs randomized to the immediate group with details about the study (e.g. where to find the decision support document in the EMR) via a link to a video of a slide-based presentation with accompanying voice-over. We also provide them with a copy of the slide set. We ask providers not discuss the decision support documents, process, or experience with other clinicians. Study team members send PCPs randomized to the wait-list control group an email with this same information 12 months later. PCPs are responsible for ordering recommended tests as they deem clinically appropriate and per patient agreement after discussion of personal preferences and medical co-morbidities.

Patients assigned to PCPs who are randomized to the immediate arm are offered the choice of completing MeTree online on their own or at the hospital with study personnel. We provide the username and password for MeTree to patients by mail, text message, or the Veterans Health Administration’s Secure Messaging system (MyHealtheVet), or multiple modes, depending on patient preference. We encourage patients to complete MeTree before their upcoming primary care visit with reminder phone calls, text messages, or secure email messages sent up to twice per week. For patients who need assistance, they may choose to complete MeTree in-person by meeting with study personnel before their scheduled appointment or at another convenient time. The number of patients requiring assistance is tracked and recorded in a study database. For patients who complete MeTree on their own, we place the provider-facing report into the patient’s EMR and alert the PCP. Patients who complete MeTree in-person receive a paper copy of the patient-facing decision support documents to share with their PCP at their appointment and the provider-facing decision support document is uploaded into the EMR. Patients in the immediate arm complete an exit survey shortly after completing MeTree (e.g. experience with program and topics discussed with their PCP) and a follow-up survey 12 months later (e.g. updated assessment of personal health screenings and genetic testing) (see Table 3). After MeTree is completed and a clinical decision support document rendered, patients may still enter new information into the system that might develop at a later time; however, any changes in their report would not be transmitted.

#### Wait-list control arm

At 10–11 months after consent of the first several patients, we contact wait-list PCPs to provide the same education (i.e. informational email and slide set) that was provided to PCPs in the immediate arm. When we disclose the randomization assignment to patients in the wait-list control arm, we inform them that we will contact them 12 months post-consent to complete MeTree. We advise these patients that there is little risk associated with waiting, as CRC takes 7–10 years to develop. During the 12-month follow-up period, neither wait-list control patients nor their providers receive further contact from study personnel.

Ten months after each wait-list control patient provides consent, we mail a reminder letter informing them about the process to complete MeTree. We also telephone these patients to assess their preference for mode of completion using the aforementioned procedures. We follow the same procedures for uploading the PCP decision support document requiring electronic signature from the provider. Wait-list control arm patients complete their exit survey and their 12-month survey at the same time (see Table 3).

All patients receive a small financial compensation after completion of MeTree (baseline or 12 months later, depending on study arm).

### Description of MeTree

MeTree is a patient-facing, web-based, FHH collection and clinical decision support platform developed in 2004 [25]. It was designed to include a patient-friendly, interactive interface for entering FHH. MeTree’s algorithms are based on current clinical guidelines used by PCPs (e.g. United States Preventive Services Task Force, National Comprehensive Cancer Network). The current version collects information on personal and FHH for 90 conditions and provides evidence-based, guideline-concordant, patient-specific recommendations for screening on 30 conditions [29]. Additional description of MeTree development, validation, and acceptability has been published [25, 29, 30].

We provide patients with a worksheet to assist them in collecting FHH from their family members before logging into MeTree. The worksheet is downloadable from the MeTree website but is also mailed to patients, depending on timing and preference. The worksheet explains the importance of FHH and guides patients to collect pertinent information from family members. Although this study focuses on CRC as the disease context for the primary outcome, we ask patients to enter their personal and FHH for all diseases included in MeTree. The MeTree decision support document includes decision support for any of the 30 conditions for which the patient may be at increased risk.

To complete MeTree, patients enter their personal and family health history directly into the web-based platform following easy-to-read prompts. Then, the MeTree program analyzes the data using existing guidelines to generate a report for the patient. MeTree also generates a provider report with citations for relevant supporting guidelines that is uploaded in the EMR for provider review.

### Measures

We are collecting patient demographics and self-reported cancer screening at baseline and 12 months after consent (see Table 3). We also assess patients’ experiences using MeTree during a brief exit survey that is administered within one month of MeTree completion for both arms. We review the EMR and administrative data to assess the remaining outcomes. Study personnel at both sites conduct chart reviews. Blinding of outcome assessors cannot be ensured because the MeTree decision support document for PCPs is part of the patient’s EMR. However, we attempt to maintain blinding to the extent possible by having study personnel complete chart abstractions without accessing the study database so that they are not directly able to see the randomization status of each patient. To decrease the risk of ascertainment bias and to standardize the abstraction process, a detailed record abstraction protocol with data dictionary is created and training was conducted by a gastroenterologist on the study team (DF).

### Outcomes

The primary outcome for this study is a binary outcome of whether risk-appropriate CRC screening referral occurred during the 12 months after consent. In order to measure this outcome, we need to know if a patient is at increased risk for CRC and the risk-appropriate recommendations for screening for that individual (i.e. the outcomes of our first aim). Thus, the primary outcome assessment follows completion of our first aim. Patients’ EMRs are reviewed for CRC-related screening referrals in the 15 months subsequent to consent. We are including an extra three months to capture any delayed uptake in recommended referrals. Specifically, clinical notes and consults are reviewed to identify referrals. For patients in both arms, risk-appropriate referral is based on the CRC risk level determined by the algorithm and recommendations from the MeTree program. Note that for patients determined to be at average risk for CRC by the MeTree program, a risk-appropriate referral may be no referral for CRC screening or surveillance.

Secondary outcomes are binary outcomes for patient completion and uptake of the CRC screening or surveillance referral, and risk-appropriate referral to genetic consultation over the 12-month follow-up period. We are examining patient uptake of CRC screening/surveillance referral for the subset of patients who received a risk-appropriate CRC screening or surveillance referral; patients who have no risk-appropriate referrals are not included in this analysis. A referral is identified by having an order in the record. Referral for CRC screening is identified as “recommended/ordered” and “completed.” “Recommended/ordered” is defined as the placement of an order for CRC screening or mention of such in the medical record, but the ordered screening is not completed. “Completed” refers to an order that was both placed and completed. We also look for the referral, actual order, and completion of risk-appropriate genetic consultation. As patient history can change over a 12-month period, when collecting self-reported screening history during the 12-month phone call, we ask patients if they have had any new relevant FHH diagnoses.

A secondary outcome for the study is baseline risk categorization for CRC in the EMR before study enrollment. We audit the EMR of all patients enrolled in the study to determine whether, at baseline, they were identified as being at increased risk for CRC based on an observation of either: (1) a diagnosis of being at increased risk for CRC (diagnosis code: V16.0); or (2) documentation of being at increased risk for CRC in the EMR. Study staff review progress notes or documentation of FHH of CRC, including the relationship and age at diagnosis or explicit statement of no FHH of CRC. This is accomplished with a text search. Initially, we are validating our search terms in a subset of 25 patients. If necessary, the terms will be adjusted to accommodate those found in the initial 25 patients and validation will be repeated on an additional 25 patients. To decrease the risk of ascertainment bias and to standardize the abstraction, a detailed record abstraction protocol with data dictionary was created and training will be conducted.

To complete follow-up and conduct both exit and 12-month surveys, we will attempt to reach patients by phone until the end of the survey window (one month after MeTree for the exit survey and 58 weeks after consent for the 12-month survey). If we cannot reach patients by phone for these surveys, we will still complete their chart review as described above.

### Data management

Our primary and secondary outcome measures are collected in an electronic chart abstraction survey using data entry/management software DatStat Illume (version 5.1 before 29 January 2019 and version 6.1 subsequently). Baseline and 12-month follow-up survey data are also captured electronically in Illume. We developed a centralized tool for capturing data across study sites electronically using a custom patient tracking application and Illume. The tracking application and Illume are housed on a secure database server behind the VA firewall with access limited to key personnel. Data entered by patients into the MeTree online tool are stored on a secure Duke website that meets VA security standards. Throughout enrollment and data collection, we monitor data quality, accuracy, and timeliness.

### Analyses

#### Primary aims

Aim 1 is to determine whether FHH collection via MeTree improves identification of patients at increased risk for CRC by comparing rates of increased risk identification in the medical record before study enrollment (baseline risk) to rates of high-risk identification by MeTree. As patients in both arms use MeTree for risk assessment, the design for this aim is a pre-/post-comparison within patient. We will test whether MeTree risk is higher than baseline risk using a McNemar’s test adjusted for clustering of patients within PCP. Note that the outcome for this aim is used in the primary outcome analysis.

Aim 2, the primary effectiveness aim, is to evaluate whether providing decision support to patients and PCPs improves risk-appropriate PCP referrals for CRC screening/surveillance and, secondarily, whether it improves patient uptake of CRC screening/surveillance and risk-appropriate referrals to genetic counseling. For the referral and uptake outcomes we will use generalized estimating equations (GEE) models to account for clustering of patients within PCP [31]. Because the primary and secondary outcomes are binary, we will use a GEE with a logit link function and an exchangeable correlation structure for each outcome. We will report 95% confidence intervals for the parameter estimates and *p* values from Wald t-tests with bias-corrected empirical sandwich estimators to maintain type I error control due to small numbers of clusters [32, 33]. Empirical standard errors are robust to misspecifications of the correlation structure.

The primary analyses for this cluster RCT will be conducted on an intent-to-treat basis. The main conclusions drawn from this trial will be based on the pre-specified primary and secondary hypotheses outlined previously and will be tested with two-sided *p* values at the standard level of 0.05 with effect size estimates and associated 95% confidence intervals reported. For both primary and secondary outcomes, stratification variables (provider type and patient age categorization) will be included in GEE models. Given that the outcomes are determined based on EMR review over a 12-month period, there should be very little missing data. The most likely scenarios to explain missing data are that a patient dies during the study or leaves the VA system before completion of the 12-month follow-up period.

We will conduct descriptive analyses of data acquired through the baseline, exit, and 12-month follow up surveys. Statistical analyses are performed using SAS for Windows (Version 9.4: SAS Institute, Cary, NC, USA) and R (http://www.R-project.org).

##### Statistical power and sample size

The sample size estimate of *n* = 250 patients with a completed MeTree assessment per arm (total *n* = 500) with 40 providers (20 in each arm) is based on 80% power, a type-I error rate of 5%, 4% loss to follow-up, and Z-test for the difference of two proportions adjusted for provider clustering. For the difference-in-proportions sample size calculation, we assumed a risk-appropriate screening referral rate for the wait-list control arm in the range of 60–70% [34]. The sample size for difference in proportions was adjusted by an intraclass correlation coefficient (ICC), a measure of “relatedness” of patients within a PCP to account for patient clustering within provider; we used an ICC of 0.02 [35, 36]. PASS 2008 [37] was used for all sample size calculations. Table 4 gives the estimated difference in proportions under the above stated assumptions that we can detect for the range of baseline referral rates in the wait-list control arm adjusted for clustering of providers and assuming a loss to follow-up of 4%.

**Table 4:** Estimated differences in CRC screening referral rates that can be detected between arms for different baseline screen referral rates with 80% power

Sample size (per arm)	Baseline CRC screening referral rate for control arm (%)	Estimated difference in CRC screening referral between arms (%)
250	60	13.2
250	65	12.7
250	70	12.0

For the secondary outcome, uptake of risk-appropriate CRC screening, our sample size is smaller as this outcome is only available for veterans that were referred for a CRC screening/surveillance test. Based on a hypothesized CRC screening/surveillance referral rate of 60% in the control arm and 73% in the immediate arm, the effective sample size after applying the 4% loss to follow-up is approximately 140 in the control arm and 180 in the immediate arm. With this effective sample size, a type-I error rate of 5%, and an ICC of 0.02, we can detect a difference of 15.6% in uptake of CRC screening test rates between arms with 80% power.

#### Secondary aims

##### Economic/cost analyses

We will also conduct a budget impact analysis (BIA) of the MeTree system to estimate the real-world financial implications of such an intervention. This analysis is conducted from the perspective of the VA healthcare system. Therefore, costs incurred by patients, such as travel or work absence to receive healthcare services, are not included. We will convert all cost figures to current dollars using the Bureau of Labor Statistics Consumer Price Index Calculator (https://data.bls.gov/cgi-bin/cpicalc.pl). Given that a horizon of 1–5 years is common for BIAs, we will use a three-year time horizon [38].

Our analyses include costs associated with the following elements: the FHH collection intervention; screening for CRC; genetic counseling; and genetic testing. These cost elements will be generated prospectively for both the immediate and wait-list control groups.

To derive intervention cost, we take an implementation perspective. The cost of the intervention will include cost of the computer required for MeTree use in clinic (if patient is met in clinic before appointment), providing the decision support documents, and the labor cost of providing assistance to patients to use MeTree and generate notes in the EMR for each patient’s PCP. We will account for the cost of training the coordinator to facilitate patient use of MeTree and entering notes in the EMR and of educating PCPs on the intervention. We will collect a sample of time required to perform each of these tasks and applying appropriate wage and fringe benefit rates to calculate labor cost. Collecting FHH through MeTree may be time-consuming and lead to longer patient visits. While we do not have a provider survey, we are conducting qualitative interviews with PCPs. During these interviews, we ask PCPs to quantify the impact of the MeTree tool on the length of their clinic visits. If PCPs report an increase or decrease in time spent with patients, then we will apply a wage-per-minute cost to the time difference to derive an incremental visit time cost. Study-related costs, such as the study coordinator’s time to consent and randomize patients, are not included. Development costs, such as designing MeTree and decision support materials, are treated as a one-time “sunk cost” that has already been incurred and are not included in our calculations.

The intervention may differentially impact utilization of healthcare services. We will derive screening costs of fecal occult blood test, flexible sigmoidoscopy, or colonoscopy from the VA’s Managerial Cost Accounting outpatient and inpatient extracts datasets. We will collect a sample of times required to provide CRC genetic counseling and wage and fringe benefit rate for genetic counselors to estimate the cost of providing CRC genetic counseling. For patients who receive genetic testing, we will apply the prevailing market rate for a genetic test at study conclusion to the analysis. Although intervention and screening costs are likely to be higher in the immediate arm, at least some of this cost may be mitigated by cost savings from CRC cases being averted. We will use estimates of the rate and cost of CRC from the literature to incorporate this cost component.

The net budget impact of the intervention is the difference in total costs incurred by patients in the immediate and wait-list control arms. We will also build on our BIA to conduct a cost-consequence analysis. The consequences of clinical interest are the additional number of appropriate referrals for genetic counseling, genetic testing, and additional cases of CRC detected due to the intervention. We will use these cost and consequence estimates from our sample to extrapolate what the budget impact and additional cases of CRC detected would be if the intervention were to be available for the entire VA healthcare system and veteran population.

##### Qualitative interviews with providers

Our study also includes a secondary aim to assess context for implementation. This aim employs qualitative methods to evaluate PCPs’ experiences using MeTree and how it affects their workflow. This information will provide important context to help interpret quantitative findings on uptake and effectiveness and additionally consider issues around operability and feasibility for future wide scale adoption and implementation. We aim to conduct 20-min semi-structured interviews by phone with 4–5 PCPs at each site. Guided by Weiner’s organizational model of implementation readiness [27, 39–41], the interviewer asks participating providers about their use of FHH in clinical decision-making (i.e. innovation-values fit), how they became aware of whether their patients completed MeTree, how they used or did not use the MeTree recommendations, how their patients reacted to the recommendations (i.e. implementation effectiveness), how MeTree affected their workflow and the time required to act on the recommendations, and any barriers to implementing MeTree recommendations (i.e. innovation-task fit). Interviews are audio-recorded and transcribed. Data will be analyzed using conventional content analysis, in which codes, or descriptive labels, will be attached to relevant passages of text in transcripts [42]. A team of two qualitative analysts code data, first working independently, and then meeting to evaluate consistency. Differences will be resolved by discussion and consensus. As is standard practice, analysis will be iterative (i.e. we code and discuss an initial transcript before moving to a second one with an updated coding scheme). The final coding scheme will be applied to all transcripts, recoding transcripts that were coded with earlier versions of the codebook as necessary. Analysts will identify themes by reviewing coded data for patterns about facilitators and barriers to inform future implementation [43].

##### Monitoring and ethics

This study does not have a data monitoring committee as no medical treatment is being provided as part of the intervention and all patients will eventually receive the intervention. There are no significant anticipated harms from this intervention; however, we track adverse events according to IRB regulations. Adverse events are not assessed systematically; they are recorded if we become aware of them (e.g. a patient mentions an illness during a conversation with a study team member). Significant protocol modifications are enacted only after IRB approval and are updated in trial registration and described in subsequent manuscripts.

## Discussion

The systematic collection of a comprehensive FHH combined with readily available, guideline-concordant decision support is crucial to promote timely, patient-centered risk assessment and management. We are conducting a trial to test the effectiveness of such an approach in the VA while gathering information that will inform future implementation.

Our study design offers distinct strengths. First, previous studies of MeTree have not included a control group, precluding a power calculation and determination of effect size. Our study adds to this previous literature as we specifically chose the inclusion of a comparator group to allow for this statistical analysis. Second, in further distinction to previous MeTree studies, we focused on a sex-neutral primary outcome allowing us to explore applicability to men and women patients alike. Third, we are conducting this study in collaboration with developers of the software (co-investigators RW, ERH, and LO), which facilitates informed conversations about risk identification and incorporation into planned analyses. Finally, as part of the shaping of the recruitment process, we sought input from patient stakeholders. We met with the Durham Center for Innovation to Accelerate Discovery and Practice Transformation’s Veteran engagement panel, who reviewed our recruitment materials and provided suggestions for tailoring study procedures to the target population.

This study also has some limitations. First, the comparator arm will not receive risk assignment until 12 months after the immediate intervention arm. This choice is justified by the ethical implications of not providing risk assessment results to patients. Fortunately, the natural progression of CRC is slow and there is little concern about this delay in overall individual patient risk. Second, because randomization takes place at the provider level, the potential patient pool is subject to system-level changes and PCP turnover due to retirement and/or new positions; however, this is expected to be minimal. Finally, all patients enrolled in this study are Veterans receiving care through the Department of Veteran Affairs. While the VA patient population tends to differ in important ways from the general United States population (e.g. fewer women, more medical comorbidities), as a national, integrated system it is comparable to other similarly structured healthcare systems [44].

## Conclusion

Results from this trial will determine the effectiveness and feasibility of incorporating patient-collected FHH and decision support on risk-identification and appropriate screening of increased-risk individuals in a large healthcare system such as the VA. Obtaining a detailed and accurate FHH is a critical step in the identification of patients at increased risk for certain cancers, including CRC. Unfortunately, FHH is rarely obtained or documented in a consistent manner that facilitates guideline-concordant, risk-appropriate care. This study will contribute to the body of literature pursuing the advancement of patient-centered, risk-appropriate care provision and the ultimate goal of reducing the morbidity and mortality of health conditions associated with genetic predisposition.

### Trial status

- Protocol version number and date: Protocol version 14, approved by IRB 1-3-2019
- Date recruitment began: 21 August 2017
- Approximate date when recruitment will complete: 31 March 2019

## Additional files

Additional file 1: World Health Organization trial registration dataset. (DOCX 15 kb)

Additional file 2: SPIRIT 2013 Checklist: Recommended items to address in a clinical trial protocol and related documents*. (DOC 122 kb)

Additional file 3: Informed consent language. (DOCX 16 kb)

## Data Availability

The datasets used and/or analyzed during the study will be maintained according to VA regulations and are not currently available for other investigators.

## Abbreviations

BIA: Budget impact analysis
CDS: Clinical decision support
CRC: Colorectal cancer
EMR: Electronic medical record
FHH: Family health history
GEE: Generalized estimating equation
ICC: Intraclass correlation coefficient
IRB: Institutional review board
PCP: Primary care provider
VA: Veterans Administration
