# Supplementary material for: An electronic family health history tool to identify and manage patients at increased risk for colorectal cancer: protocol for a randomized controlled trial
Source: Trials. 2019 Oct 7;20:576. doi: 10.1186/s13063-019-3659-y (PMC6781340; doi:10.1186/s13063-019-3659-y)
Supplement: Supplementary file 1 — World Health Organization trial registration dataset. (DOCX 15 kb) [file 13063_2019_3659_MOESM1_ESM.docx]

Appendix A. World Health Organization Trial Registration Data Set

| **Data category** | **Information** |
| --- | --- |
| Primary registry and trial identifying number | ClinicalTrials.gov NCT02247336 |
| Date of registration in primary registry | September 25, 2014 |
| Secondary identifying numbers | IIR 12-378 |
| Source(s) of monetary or material support | VA Office of Research and Development |
| Primary sponsor | VA Office of Research and Development |
| Secondary sponsor(s) | NA |
| Contact for public queries | Corrine I. Voils, PhD William S. Middleton Memorial Veterans Hospital, Madison, WI |
| Contact for scientific queries | Corrine I. Voils, PhD William S. Middleton Memorial Veterans Hospital, Madison, WI |
| Public title | Impact of Family History and Decision Support on High-risk Cancer Screening |
| Scientific title | Impact of Family History and Decision Support on High-risk Cancer Screening |
| Countries of recruitment | United States |
| Health condition(s) or problem(s) studied | Colorectal cancer |
| Intervention(s) | Participants will enter their family health history information into MeTree, patients and providers will receive a decision support document and pedigree |
| Key inclusion and exclusion criteria | Inclusion Criteria:  Primary care provider inclusion criteria:   - primary care physician, - physician assistant, or nurse practitioner; - at least one half-day of primary care clinic per week.   Patient inclusion criteria:   - assigned to an enrolled PCP; - English as preferred language; - no plans to relocate or leave the VA system in the next 12 months; - at least one primary care appointment in the 18 months prior to enrollment; - upcoming PCP appointment with assigned PCP; - aged 40-64 years; no previous history of colorectal cancer or adenomatous polyps or inflammatory bowel disease; - no endoscopy within previous 4 years; some knowledge of family health history   Exclusion Criteria:  n/a (contained within inclusion criteria) |
| Study type | Allocation: Randomized Intervention Model: Parallel Assignment Masking: None (Open Label) Primary Purpose: Prevention |
| Date of first enrolment | August 1, 2017 |
| Target sample size | 600 |
| Recruitment status | Recruiting by invitation |
| Primary outcome(s) | Provider referral for risk-appropriate colorectal cancer screening |
| Key secondary outcomes | - Patient uptake of risk-appropriate colorectal cancer screening - Referral for genetic consultation |
