# Supplementary material for: An electronic family health history tool to identify and manage patients at increased risk for colorectal cancer: protocol for a randomized controlled trial
Source: Trials. 2019 Oct 7;20:576. doi: 10.1186/s13063-019-3659-y (PMC6781340; doi:10.1186/s13063-019-3659-y)
Supplement: Supplementary file 3 — Informed consent language. (DOCX 16 kb) [file 13063_2019_3659_MOESM3_ESM.docx]

**Appendix B. Informed Consent Language**

(Note that this study required verbal informed consent. Below is a transcript of the language used to conduct informed consent via phone.)

**This telephone consent will take the place of a written consent to be in the study. Over 3 1/2 years, we will enroll and collect data from up to 600 participants in this study. Your participation will be limited to entering your family health history information into MeTree on your own or in person and completing a survey. The survey asks about screening that you have had for cancer and other health problems. We will look in your medical record to see if you receive any cancer screening in the 12 months after you use MeTree.**

You are not required to take part in this study: your participation is entirely voluntary.

You can refuse to participate now or you can withdraw from the study at any time after giving your consent.  This will not interfere with your regular medical treatment.

If you want to withdraw from the study, you can do this by writing the Principal Investigator. Instructions for doing so will be on the HIPAA Authorization form that we provide to you.

You may not personally be helped by being in this study, but your participation may lead to knowledge that will help others.

If you are a patient and decide you do not want to be in this study, your decision will not affect your treatment in any way. You will continue to receive usual care for high blood pressure from your doctor, whether or not you are in this study.

If during the study any information reveals depression or other major clinical findings, your primary physician will be notified immediately.

If results of this study are reported in medical journals or at meetings, you will not be identified by name, by recognizable photograph, or by any other means without your specific consent.

Your medical records will be maintained according to this medical center’s requirements. However, there is a possibility that the Office for Human Research Protections (OHRP), the Office of Research Oversight (ORO), the Institutional Review Board (IRB), or the Department of Veterans Affairs may inspect the records.

There will be no cost to you for any of the treatment or testing done as part of this research study.

Some veterans are required to pay co-payments for medical care and services provided by VA.  These co-payment requirements will continue to apply to medical care and services provided by VA that are not part of this study.

Eligibility for medical care is based upon the usual VA eligibility policy and is not guaranteed by participation in a research study.

In case of illness or physical injury resulting from participation in this study, you are entitled to medical care and treatment, except if the illness or injury is due to noncompliance (not following study instructions) by you.  This care may be provided by the VAMC or arrangements may be made for contracted care at another facility.

The VAMC has not set aside compensation payable in the event of physical injury or illness resulting from participation in this study.

**In case there are questions, concerns, or complaints regarding this research study, you can call the study Principal Investigator or one of the study doctors. I will give you their phone numbers at the end of this call so you can write them down. If any medical problems occur in connection with this study the VA will provide care.**

Further information about compensation and medical treatment may be obtained from the medical administration service at this VA medical center. I will give you their number at the end of the phone call.

If you have questions about the research or your rights as a research subject, would like to obtain information, offer input, or have other concerns or complaints, you may contact the administrative officer of the research service. I will also give you that number at the end of this call.

Do you have any questions? *(address)*

As a reminder, your family history data will be entered into the MeTree program. This tool was developed and lives on a Duke computer behind the Duke firewall and meets all VA security standards. Identifying information, such as your name or address, will not be entered into the program. Your family history data will be combined with family history data collected from other patients and maintained indefinitely by Duke for future research about how diseases are passed down in families. All other data collected during this study, including identifying information and any health data abstracted from your VA medical record, will be kept in VA files only. If you decide not to bank your family health history information at Duke, then you cannot take part in this study.

Do you agree that your rights as a research subject have been explained to you and you voluntarily consent to participate in this study?
